# Genetic and Environmental Influences on Dental Arch Shape During Development: A Longitudinal Twin Study

**DOI:** 10.1111/ocr.70162

**Published:** 2026-07-01

**Authors:** Jamal Giri, Michelle Bockmann, Alan Brook, Angela Gurr, Lyle Palmer, Toby Hughes

**Affiliations:** ^1^ Adelaide Dental School, Faculty of Health and Medical Sciences The University of Adelaide Adelaide South Australia Australia; ^2^ School of Public Health, Faculty of Health and Medical Sciences The University of Adelaide Adelaide South Australia Australia; ^3^ Australian Institute of Machine Learning The University of Adelaide Adelaide South Australia Australia

**Keywords:** arch shape, dental arch, environment, genetics, heritability, twins

## Abstract

**Objective:**

This study aimed to determine the relative contributions of genetic and environmental factors to phenotypic variations of dental arch shape from the primary to permanent dentition stages.

**Materials and Methods:**

Maxillary and mandibular digital models of 188 twin pairs (90 monozygotic and 98 dizygotic) in the primary dentition stage, tracked longitudinally through the mixed and permanent dentition stages, were examined. Dental landmarks were recorded at the incisal edges of incisors and the cusp tips of canines and posterior teeth in both arches in MeshLab. Arch shape variation was analysed using Procrustes superimposition and principal component analysis, then partitioned into genetic and environmental variance components using genetic structural equation modelling.

**Results:**

The first three principal components were meaningful, explaining 42%–54% of the phenotypic variation in dental arch shape from the primary to permanent dentition stages. The arch depth‐to‐width ratio (principal component 1) greatly influenced arch shape in both maxillary and mandibular arches across all dentition stages. Other components included canine position, displacement and rotation of incisors and posterior teeth and the steepness of the curve of Spee. An AE model incorporating additive genetic (A) and non‐shared environmental (E) components best explained the phenotypic variances in dental arch shape, with narrow‐sense heritability estimates ranging from 0.60 to 0.84 for the first three principal components.

**Conclusions:**

The dental arch shape was predominantly influenced by additive genetic and non‐shared environmental factors throughout development, with moderate to high heritability estimates across all dentition stages.

## 1 Introduction

The dental arch form has substantial clinical importance in orthodontics, as arch morphology affects both diagnosis and treatment planning. Change in arch form during orthodontic treatment is associated with relapse and periodontal damage [1]. Several studies have extensively investigated arch form, in attempts to determine an ideal shape that could be universally applied to all orthodontic patients [2, 3]. These studies have used various mathematical equations and curves, such as ellipses, conic sections, polynomial functions, catenary curves and Fourier series to define a universal arch shape, but without success [4, 5].

Considerable variation in arch shape is observed among individuals and across populations [6, 7, 8]. While the basic shape of the dental arch is established in utero, its variability is influenced by several postnatal factors, including the growth of underlying basal bone, the eruptive paths of teeth and the forces exerted by perioral soft tissues [9, 10]. This variation can be largely attributed to the complex interactions between genetic and environmental factors at multiple levels (molecular, cellular and tissue levels) during dental arch development [11].

Family studies, such as twin pairs, sibships and parent‐offspring triads, have been used to explore genetic influence on human traits. Twin studies, in particular, have been crucial in uncovering genetic and environmental influences on dentofacial development [12, 13]. The classical twin model is the most powerful design for estimating genetic and environmental contributions to phenotypic variability by comparing monozygotic (MZ) and dizygotic (DZ) twins raised in the same family. MZ twins are genetically near identical, while DZ twins share 50% of their genetic variation on average (the same as any siblings with the same parents). By comparing the phenotypic data of twins raised in the same environment, the total variance of a trait can be statistically decomposed into genetic and environmental variances [14].

Despite substantial research on dental arches among twins, the emphasis has largely been on arch dimensions, with shape receiving less attention [15]. While some twin studies have reported varying genetic influences on arch shapes, they relied on two‐dimensional (2D) assessments of dental arches using standardised photographs of dental models [16, 17, 18]. The 2D photographic assessment fails to capture the full complexity of the arch shape of three‐dimensional (3D) dental models. Geometric morphometrics (GMM) of 3D dental models offers a more comprehensive understanding of dental arch shape, capturing multivariate geometric data often missed by 2D assessments [19]. A twin study using GMM on 3D dental models of the permanent dentition stage found that maxillary and mandibular arch shapes exhibited moderate to high heritability in adults [20]. The influence of genetic and environmental factors on arch shape during the primary and mixed dentition stages and how these factors vary with time is not fully understood. Therefore, a longitudinal study of 3D dental models of twins from the primary to the permanent dentition stages using GMM is necessary to address this gap in our understanding of the genetic and environmental influences on arch shape during development.

The present study aimed to characterise dental arch shape using principal components derived from geometric morphometric analysis of dental arches across primary, mixed and permanent dentition stages; quantify the relative contributions of additive genetic (A), shared environmental (C) and non‐shared environmental (E) factors to variation in these shape components; and examine how these contributions vary across dentition stages. We hypothesised that there are no significant differences in genetic and environmental influences on dental arch shapes across different dentition stages.

## 2 Materials and Methods

### 2.1 Study Population

Dental models from 300 twin pairs enrolled in a longitudinal cohort study in South Australia were screened for inclusion in this retrospective analysis. Dental models and associated metadata were collected longitudinally between 1995 and 2006 [21]. The twins were of European ancestry without any craniofacial anomalies. Dental models of the twins were obtained longitudinally by making alginate impressions of maxillary and mandibular arches at primary (5.8 ± 0.7 years), mixed (9.4 ± 0.8 years) and permanent (14.2 ± 0.8 years) dentition stages of development between 1995 and 2006. Twin zygosity was determined by genotyping up to six highly polymorphic genetic markers (FES, vWA31, F13A1, THO1, D21S11 and FGA) using DNA extracted from buccal cells [22]. Ethical approval was granted by the Human Research Ethics Committee of the University of Adelaide (Reference number: H‐2023‐060).

The dental models of the twins were screened using the following exclusion criteria:

1. History of orthodontic treatment
2. Damaged or poor‐quality models
3. Presence of dental variations such as missing or supernumerary teeth and ectopic eruption of teeth

After applying these criteria, dental models from 188 twin pairs (90 MZ and 98 DZ) in the primary dentition stage, 181 twin pairs (88 MZ and 93 DZ) in the mixed dentition stage and 134 twin pairs (69 MZ and 65 DZ) in the permanent dentition stage were included in the study.

Dental models were scanned using a 3D scanner (3Shape, E4, Copenhagen, Denmark) with a resolution of 4 μm. The scanned digital models were imported into MeshLab (ISTI‐CNR, Pisa, Italy, version 2022.02) for landmark digitisation. Cartesian coordinates of mesial and distal incisal edges of incisors and cusp tips of canines and all posterior teeth of both maxillary and mandibular casts were recorded (Figure 1).

**FIGURE 1:**
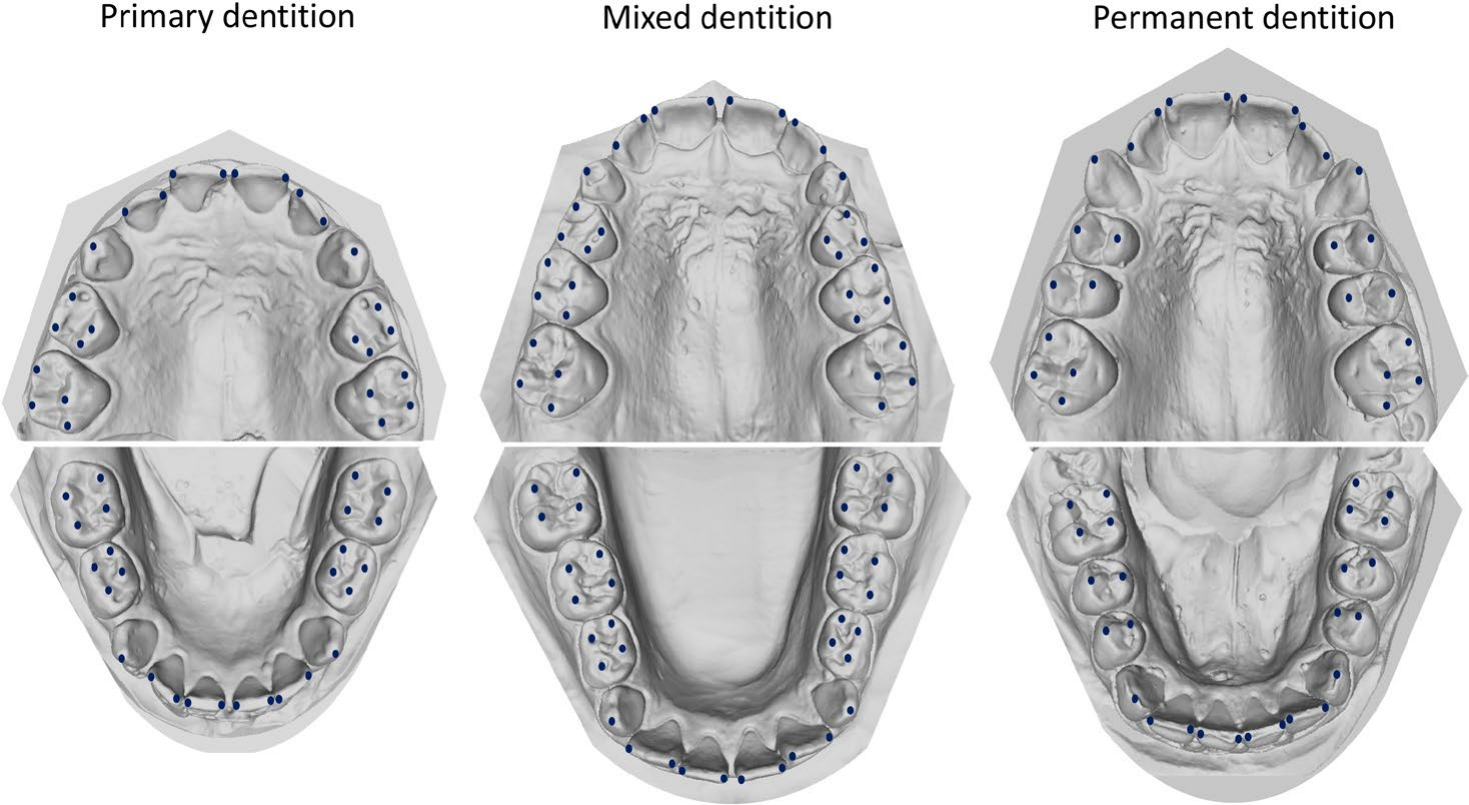
Digitised landmarks on the maxillary and mandibular dental models.

### 2.2 Method Error

All dental models were landmarked by a single examiner (J.G.). For intra‐examiner reliability assessment, 60 randomly selected maxillary and mandibular dental models (20 each of primary, mixed and permanent dentition stages) were re‐landmarked by the same examiner after 4 weeks. Another examiner (A.G.) re‐landmarked the same 60 dental models for inter‐examiner reliability assessment. Method error was assessed using Procrustes ANOVA and reported as a percentage of the total shape variance.

### 2.3 Geometric Morphometrics and Principal Component Analysis (PCA)

Generalised full Procrustes superimposition was applied to the dental arch coordinates to standardise their size, position and orientation while retaining their shape [23]. This process consists of three main steps—translation, scaling and rotation—which eliminate any non‐shape‐related differences [23]. The resulting Procrustes coordinates retain only shape‐related information, allowing for the comparison of dental arch shapes between twins.

PCA was applied to Procrustes coordinates to reduce the complex data into principal components [24]. PCAs identify linear combinations of coordinates that explain variation in arch shapes. Meaningful principal components were visualised using thin‐plate splines. The Procrustes coordinates were projected onto the principal component axes and the resulting principal component scores were used for subsequent genetic modelling. To evaluate the influence of sex and zygosity on dental arch shape variation, a Procrustes ANOVA was performed using Procrustes coordinates. Statistical significance was determined using Goodall's *F*‐test [25].

### 2.4 Genetic Analysis

To estimate the relative contributions of genetic and environmental factors to maxillary and mandibular arch shapes, genetic structural equation modelling (SEM) was performed at three different stages of dental development using the OpenMx package in R [26]. Genetic SEM operated under the usual assumptions of classical twin models [27]. The sources of variation considered in the model were: additive genetic variation (A), non‐additive genetic variation (D), shared environmental variation (C) and non‐shared environmental variation (E) (Figure 2). ACE and ADE models were fit to the principal component scores of the twin pairs to estimate the sources of phenotypic variance of dental arch shape. ACE and ADE models were fit separately because C and D components cannot be estimated simultaneously in twins raised together [28]. Full sex‐limitation models were used to estimate genetic and environmental influences in males and females separately when sexual dimorphism in arch shapes was observed [29].

**FIGURE 2:**
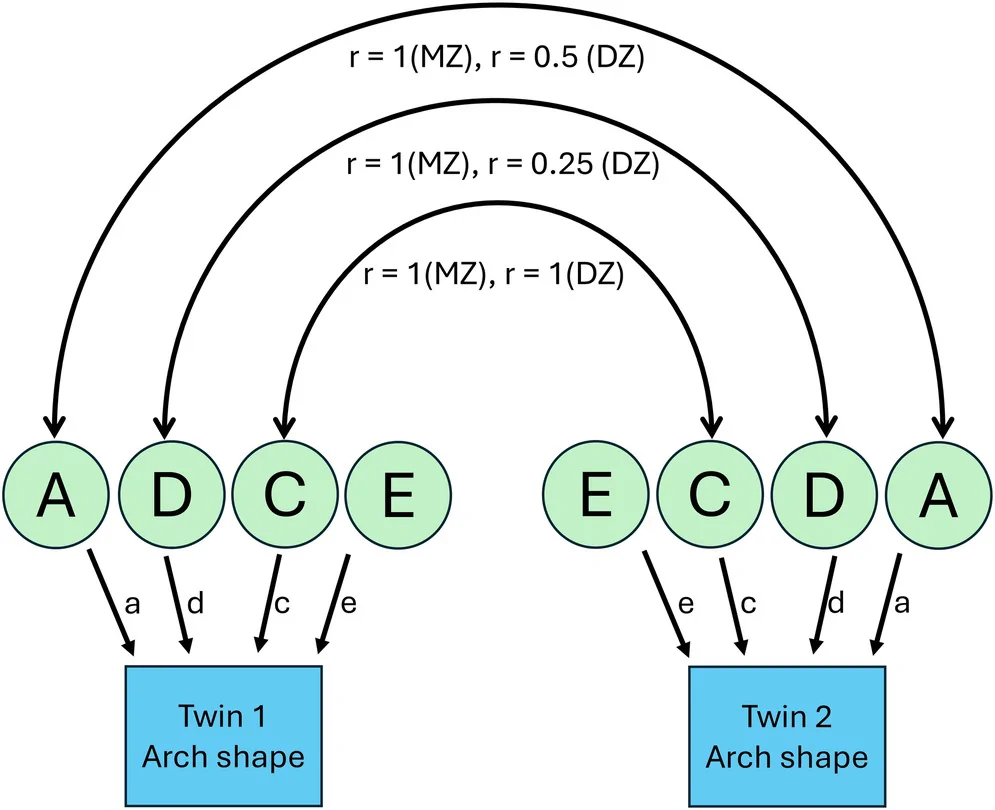
Path diagram depicting potential factors influencing twin pairs' phenotypes (arch shapes). The latent factors potentially influencing the traits are additive genetic (A), non‐additive genetic (D), shared environmental (C) and non‐shared environmental (E) factors. The path coefficients (a, d, c and e) indicate the relative contribution of each factor. The double‐headed arrows indicate the correlation (*r*) between latent factors among monozygotic (MZ) and dizygotic (DZ) twin pairs.

To evaluate the goodness‐of‐fit of full models with their nested sub‐models (e.g., ACE vs. AE) *χ* ^2^ likelihood ratio tests were used [29]. A simpler model was rejected only if it showed a significant loss of fit (*p* < 0.05). For comparisons of non‐nested models (e.g., AE vs. CE), Akaike's Information Criterion (AIC) was used, with a lower AIC value indicating a better fit. For the best‐fitting model, narrow‐sense heritability (*h* ^2^_N_) estimates were calculated for each principal component of arch shape as the ratio of additive genetic variance to total phenotypic variance [30]. All statistical analyses were performed in the R programming environment, with a significance level set at 0.05.

## 3 Results

The intra‐examiner mean random error, derived from 60 re‐landmarked dental models and expressed as a percentage of total shape variance, was 0.3% for the maxillary arch and 0.6% for the mandibular arch, while the inter‐examiner error was 2.4% for the maxillary arch and 2.7% for the mandibular arch.

Sample distributions in the shape space following Procrustes superimposition at primary, mixed and permanent dentition stages are presented in Figure 3. The first seven principal components accounted for nearly 69%, 61% and 65% of the total shape variability of the maxillary arch in the primary, mixed and permanent dentition stages, respectively. Similarly, the total shape variability of the mandibular arch in the primary, mixed and permanent dentition stages accounted for by the first seven principal components was around 65%, 63% and 65%, respectively (Table 1). Based on Scree plots, the first three principal components of both arches were considered meaningful.

**FIGURE 3:**
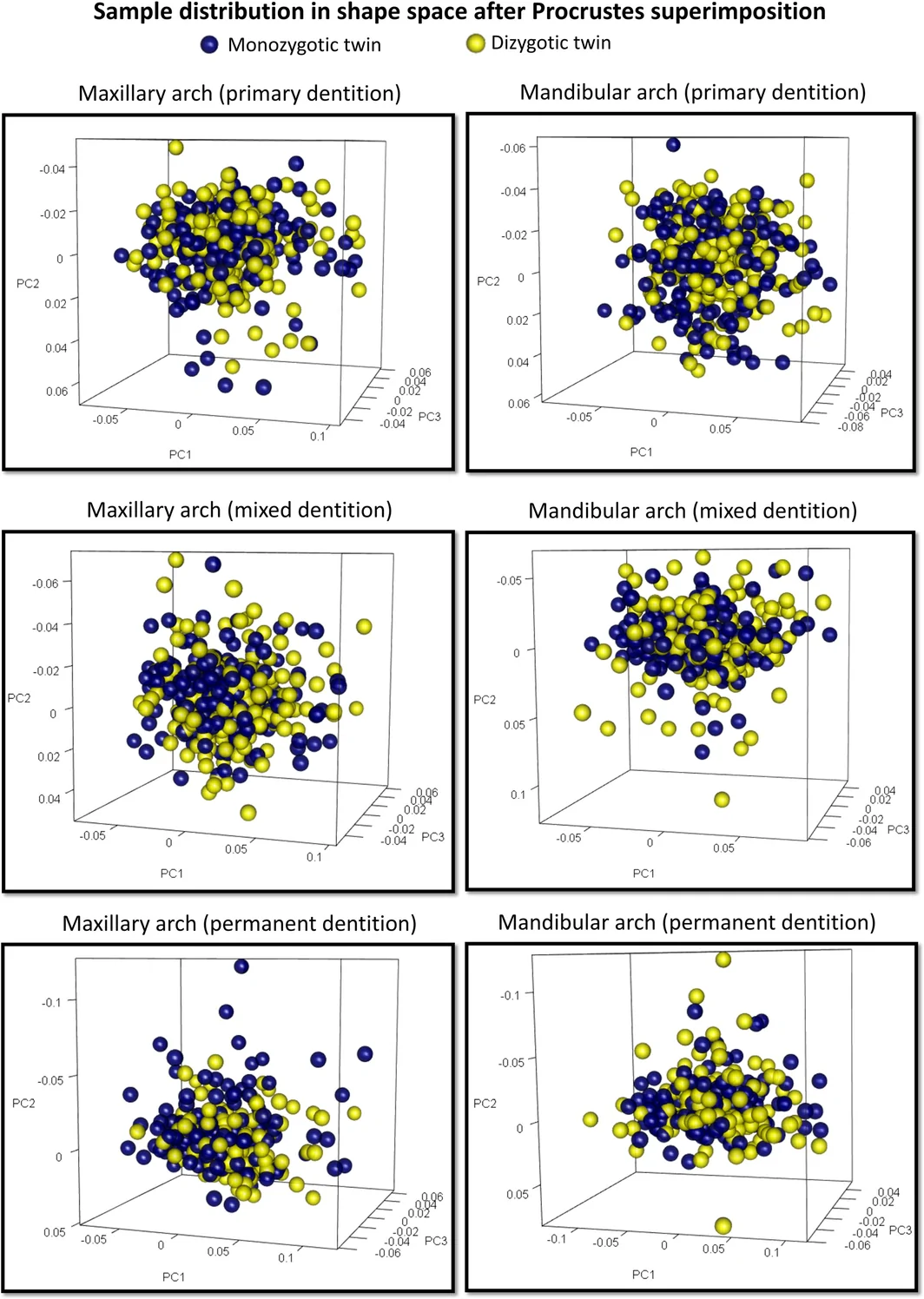
Sample distribution in shape space after Procrustes superimposition.

**TABLE 1:** Percentage of total shape variability of dental arches explained by the first seven principal components.

Dental arch	Dentition stage	PC1	PC2	PC3	PC4	PC5	PC6	PC7	Sum
Maxillary	Primary	37.8%	9.9%	6.6%	4.8%	3.9%	3.4%	2.6%	69.0%
	Mixed	25.7%	9.1%	6.9%	5.5%	5.2%	4.9%	3.6%	60.9%
	Permanent	27.3%	11.2%	7.9%	6.9%	4.7%	3.9%	2.7%	64.6%
Mandibular	Primary	28.6%	12.8%	7.0%	4.8%	4.7%	4.0%	3.2%	65.1%
	Mixed	21.6%	14.5%	7.5%	5.7%	5.3%	4.7%	3.5%	62.8%
	Permanent	31.8%	10.3%	6.7%	4.9%	4.0%	3.6%	3.5%	64.8%

Abbreviation: PC, principal component.

The first principal component (PC1) described the ratio of arch depth to arch width in both maxillary and mandibular arches across all dentition stages (Figure 4). PC2 represented the labial‐palatal position of canines in the maxillary arch. However, in the mandibular arch, PC2 captured different features depending on the dentition stage; PC2 described the rotation of incisors and labial‐lingual displacement of canine in the primary dentition while in the mixed and the permanent dentition, PC2 described the labial‐lingual displacement of incisors and mesiodistal displacement of posterior teeth in the mandibular arch (Figure S1). In the maxillary arch, PC3 described the labial‐palatal and vertical displacement of incisors in all dentition stages. In the mandibular arch, PC3 described the labial‐lingual and vertical displacement of incisors in the primary dentition but captured the steepness of the curve of Spee in the mixed and permanent dentition stages (Figure S2).

**FIGURE 4:**
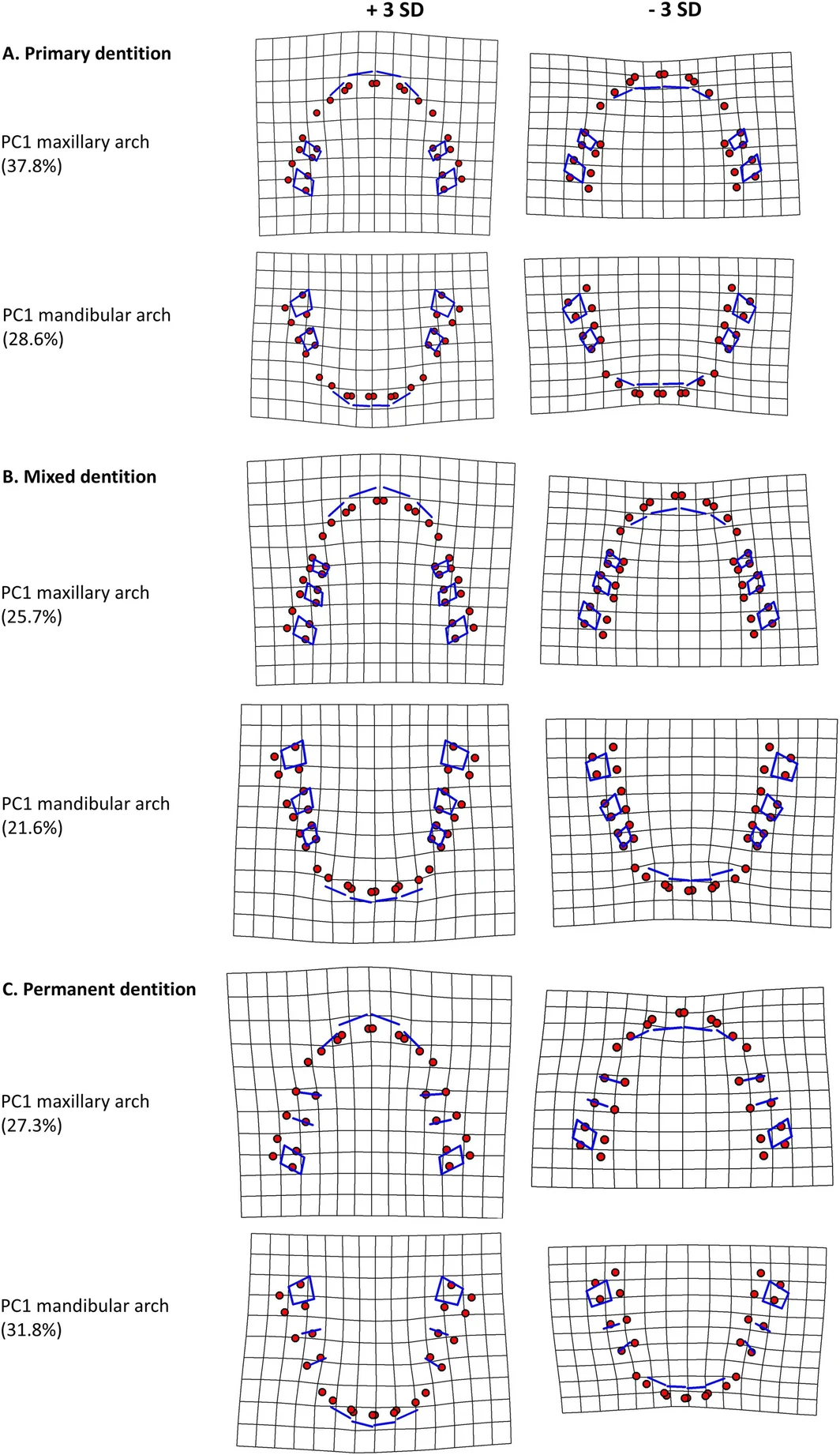
Thin plate spline (TPS) grids illustrating the average deformation of dental arches across different dentition stages for the first principal component (PC1). TPS grids show deformation at ±3 standard deviations (SD), with the consensus shape represented by red dots and the ±3 SD configurations outlined in blue. At +3 SD, arch depth increases while arch width decreases, resulting in a higher arch depth‐to‐width ratio. In contrast, at −3 SD, arch depth decreases while arch width increases, resulting in a lower arch depth‐to‐width ratio. (A) Primary dentition, (B) mixed dentition and (C) permanent dentition.

No significant differences in maxillary or mandibular arch shapes were found between MZ and DZ twins across all three dentition stages. In the primary dentition, no significant difference in maxillary or mandibular arch shape was observed between males and females. However, in the mixed and permanent dentition stages, sexual dimorphism was observed in both maxillary and mandibular arch shapes (*p* < 0.05) (Tables S1 and S2).

A model comprising additive genetic and non‐shared environmental variance components (AE model) best explained observed phenotypic variances of arch shape except for PC3 of the mandibular arch in the primary dentition, in which a model with shared and non‐shared environmental variance components (CE model) was the most parsimonious. There was no evidence of non‐additive genetic variance influencing arch shape across the dentition stages. The full sex‐limitation genetic models did not fit the data better than simpler models without sex differences across the dentition stages, suggesting similar genetic and environmental variance components for arch shapes between males and females, with no significant sex differences. Narrow‐sense heritability estimates for the first three principal components ranged between 0.60 and 0.84 (Table 2).

**TABLE 2:** Estimates of variance components and 95% confidence intervals for the first three principal components.

Principal components	Model	Variance components						
		*A*	95% CI	*C*	95% CI	*E*	95% CI	*h* ^2^_N_
*Primary dentition*								
Maxillary PC1	AE	0.74	0.64–0.81	—	—	0.26	0.19–0.35	0.74
Maxillary PC2	AE	0.76	0.68–0.82	—	—	0.24	0.17–0.31	0.76
Maxillary PC3	AE	0.71	0.62–0.78	—	—	0.29	0.21–0.37	0.71
Mandibular PC1	AE	0.76	0.68–0.83	—	—	0.24	0.17–0.32	0.76
Mandibular PC2	AE	0.77	0.68–0.82	—	—	0.23	0.17–0.31	0.77
Mandibular PC3	CE	—	—	0.57	0.46–0.65	0.43	0.34–0.53	—
*Mixed dentition*								
Maxillary PC1	AE	0.75	0.66–0.81	—	—	0.25	0.18–0.34	0.75
Maxillary PC2	AE	0.84	0.77–0.88	—	—	0.16	0.11–0.23	0.84
Maxillary PC3	AE	0.66	0.54–0.74	—	—	0.34	0.25–0.45	0.66
Mandibular PC1	AE	0.81	0.73–0.85	—	—	0.19	0.14–0.26	0.81
Mandibular PC2	AE	0.82	0.72–0.87	—	—	0.18	0.12–0.27	0.82
Mandibular PC3	AE	0.74	0.64–0.81	—	—	0.26	0.18–0.35	0.74
*Permanent dentition*								
Maxillary PC1	AE	0.82	0.74–0.87	—	—	0.18	0.12–0.25	0.82
Maxillary PC2	AE	0.75	0.64–0.81	—	—	0.25	0.18–0.35	0.75
Maxillary PC3	AE	0.60	0.43–0.72	—	—	0.40	0.27–0.56	0.60
Mandibular PC1	AE	0.83	0.76–0.88	—	—	0.17	0.11–0.23	0.83
Mandibular PC2	AE	0.69	0.56–0.78	—	—	0.31	0.21–0.43	0.69
Mandibular PC3	AE	0.70	0.56–0.79	—	—	0.30	0.20–0.43	0.70

Abbreviations: *A*: additive genetic variance, *C*: shared environmental variance, CI: confidence interval, *E*: non‐shared environmental variance, *h* ^2^_N_: narrow‐sense heritability, PC1: principal component 1, PC2: principal component 2, PC3: principal component 3.

## 4 Discussion

This longitudinal study examined dental arch shapes of twins for the first time from primary to permanent dentition stages using GMM and genetic SEM to quantify genetic and environmental influences on their development. Three meaningful principal components influencing the variation in maxillary and mandibular arch shapes were identified. SEM analyses revealed that additive genetic and non‐shared environmental factors primarily influenced maxillary and mandibular arch shape variations from primary to permanent dentition stages.

Our study found that the first three principal components accounted for 42%–54% of the morphological variation in dental arch shape across the primary to permanent dentition stages. The finding that the first three principal components account for only around 50% of the variance suggests that dental arch shape variation is complex and multifactorial rather than being driven by a few dominant patterns, unlike the arch size, where the first few principal components are dominated by allometry, capturing nearly all the variance. The observed wide variation in dental arch shape explains why commercially available orthodontic archwires often do not conform to the natural shapes of dental arches in patients [31].

PC1, which accounted for the largest proportion of shape variance in both maxillary and mandibular arches, captured the arch depth to arch width ratio across all dentition stages. While PC1 explained 38% of shape variance in the maxillary arch and 29% in the mandibular arch in the primary dentition stage, its contribution decreased in the mixed dentition stage, accounting for only 26% and 22% of variance in the maxillary and mandibular arches, respectively. This suggests that the contribution of the arch depth‐to‐width ratio to the overall arch shape fluctuates during development. However, in the absence of similar longitudinal studies, these findings could not be confirmed, emphasising the need for more research to understand developmental changes in arch morphology. In the permanent dentition stage, PC1 accounted for 27% and 32% of shape variance in the maxillary and mandibular arches, respectively, which is consistent with the findings from earlier studies [20, 32].

Our finding that additive genetic and non‐shared environmental factors best explained the phenotypic variance explained by PC1 is consistent with the findings of Lin et al. [20], with heritability estimates above 0.7 in both arches. Earlier studies on arch shape [10, 33] also found significant additive genetic influences on the arch depth‐to‐width ratio, but reported heritability estimates that were lower than those observed in the current study. This could be due to differences in populations and methods of heritability estimation. Richards et al. [16] reported that genetic factors influenced the maxillary arch shape more strongly than the mandibular arch in the permanent dentition. In contrast, our study found a strong additive genetic influence (*h* ^2^_N_ > 0.8) on both arches.

PC2, which captured the labial‐palatal position of canines in the maxillary arch, accounted for around 9%–11% of arch shape variance and was under the additive genetic and non‐shared environmental influences across the dentition stages. In the maxillary arch, the position of the canines was the second‐most significant shape‐defining feature after the arch depth to arch width ratio. It is possible that labial or palatal displacement of canines changes the taper of the maxillary arch in the anterior region, leading to shape variability. Our finding is consistent with those of Lin et al. [20]. In contrast to our finding, Papagiannis and Halazonetis [32] reported that the shape variation of the maxillary arch in the permanent dentition was mostly influenced by the ectopic position of canines in the vertical plane. In our study, PC2 in the mandibular arch accounted for around 10%–15% of arch shape variation and captured different features according to dentition stages, which included labial‐lingual displacement of canines and rotation of incisors in the primary dentition. However, in the mixed and the permanent dentitions, PC2 described the labial‐lingual displacement of incisors and mesiodistal displacement of posterior teeth. The fact that PC2 consistently represented only labial‐palatal displacement of the maxillary canines across all dentition stages, while capturing multiple features in the mandibular arch, suggests that the mandibular arch has more sources of variation. Future studies should explore whether the mandibular arch exhibits more shape variation than the maxillary arch.

PC3 described the labial‐palatal and vertical displacement of incisors in both arches except for the mandibular arch in the mixed and the permanent dentitions, where it described the steepness of the curve of Spee. PC3 accounted for around 6%–8% of the shape variance and was mostly under additive genetic and non‐shared environmental influence. However, the phenotypic variance of PC3 of the mandibular arch in the primary dentition was found to be under shared and non‐shared environmental influences, suggesting that early life environmental factors influence the position of mandibular incisors and anterior shape of the mandibular arch. This could be attributed to sucking habits (pacifiers or digits) common among children during that developmental stage. However, it is unclear why the maxillary incisors were not influenced by environmental factors as much as the mandibular incisors. Smaller roots of the mandibular incisors may offer less resistance to displacement, making them more susceptible to environmental influences from functional and habitual activities [34]. The non‐shared environmental component (E) represents influences unique to each individual, such as oral habits, dietary patterns and oral hygiene practices, including measurement error. As detailed data on these variables were not available for the twin cohort, the E component could only be interpreted as an aggregate measure without attribution to specific exposures. Future studies incorporating directly measured environmental variables may enable more precise characterisation of these effects and strengthen etiological inferences. Environmental influence in the anterior region of the mandibular arch in the primary dentition is an interesting finding and should be further investigated.

We found that dental arch shape was under strong additive genetic determination overall across the primary to the permanent dentition stages. Heritability estimates of PC1, which explained the largest variance in arch shape, increased from 0.74 to 0.82 for the maxillary arch and 0.76–0.83 for the mandibular arch from primary to permanent dentition stages. It suggests that early life environmental factors are crucial, while genetic influence on arch shape increases with age. While the estimates for PC2 and PC3 fluctuated across the dentition stages, the estimates were still in the range of 0.60–0.83. Heritability estimates for arch shape were comparable to those for arch size reported in previous studies on the primary and permanent dentition stages [22, 35], suggesting that both arch size and shape are under similar genetic influences. However, in the absence of a similar study in mixed dentition, our findings could not be confirmed.

Our study did not find any significant difference in maxillary and mandibular arch shapes between males and females in the primary dentition stage. However, there were statistically significant differences in arch shapes between males and females in the mixed and permanent dentition stages. Earlier studies in the permanent dentition stage have reported conflicting findings regarding sexual dimorphism in arch shape [20, 32]. Our findings align with those of Lin et al. [20] who also investigated twins of European ancestry and reported statistically significant differences in arch shape between the sexes. However, no meaningful sex difference in genetic or environmental influences on arch shape was detected in the full‐sex limitation model, suggesting that similar genetic and environmental factors influence arch shape in males and females. Although our sample was relatively large for a twin study, it might be underpowered to detect subtle sex‐specific genetic effects. Therefore, the absence of significant sex differences in arch shape should be interpreted with caution. Future research should also explore the role of epigenetic factors in subtle sex‐related arch shape variations in mixed and permanent dentition stages that could not be explained by genetic or environmental factors in this study.

Mossey [36] highlighted that the success of orthodontic therapy ultimately depends on clinicians' ability to ascertain the relative contributions of genetic and environmental factors on malocclusion. Using genetic SEM, we estimated the relative contributions of genetic and environmental factors to arch shape development. These findings could inform orthodontic treatment planning. The longitudinal nature of our study may be useful for optimising the timing of orthodontic intervention. As an orthodontic intervention is considered an environmental factor [37], timing the intervention when there is a stronger environmental influence or a weaker genetic influence could result in more favourable outcomes.

Preserving the original arch form, particularly, in the mandibular arch, is considered crucial for the stability of orthodontic outcomes [38]. Principal components derived from geometric morphometric analysis of dental arches capture distinct modes of variation in dental arch shape, including the arch depth to arch width ratio and the labiolingual positions of the canines and incisors. These shape components are clinically relevant, as they influence tooth size–arch length relationships and contribute to transverse discrepancies. Given a strong genetic influence on dental arch shape, particularly, on the arch depth to arch width ratio (PC1), in the permanent dentition stage, orthodontic intervention can only alter the shape within a certain limit, beyond which there is a risk of relapse. Based on our findings, both maxillary and mandibular arch shapes have strong additive genetic determinants and should be preserved as much as possible during orthodontic therapy for stable outcomes. However, in cases such as developing Class II malocclusion, a narrow maxillary arch form is evident in the primary dentition stage and has to be altered substantially [39]. Our finding that genetic influence on arch shape becomes stronger with age suggests that any intervention in arch shape should be undertaken early when indicated for the individual patient. This puts preventive and interceptive orthodontics in the spotlight because the primary or early mixed dentition stage could be the optimum time to make stable changes in dental arch shape [40].

A unique strength of the current study is the application of GMM and genetic model‐fitting techniques in a longitudinal twin sample, which provides a robust framework for exploring genetic and environmental influences on dental arch shape development. However, epigenetic influences on arch shape could not be estimated. Each dentition stage was analysed separately in this study; future studies should incorporate longitudinal modelling approaches to account for repeated measures within individuals. Furthermore, the inclusion of twins of European ancestry may restrict the generalizability of the findings to other populations. As genetic and environmental influences on traits are population‐specific, future research should investigate their role in different populations.

## 5 Conclusions

1. The arch depth to arch width ratio had the greatest influence on arch shape in both the maxillary and mandibular arches across all dentition stages. Other components defining the arch shape were the position of canines, the displacement and rotation of the incisors and posterior teeth and the steepness of the curve of Spee, which varied among the arches and across dentition stages.
2. Additive genetic and non‐shared environmental factors best explained the variations in dental arch shape with moderate to high heritability estimates across all dentition stages.
3. For arch depth to arch width ratios, the heritability estimates increased gradually from the primary to the permanent dentition stages in both arches, suggesting (a) early life environmental factors are important and (b) increasing genetic expression with age. However, heritability estimates of other arch shape features fluctuated across the dentition stages.

## Author Contributions

**Jamal Giri:** conceptualization, investigation, methodology, data curation, formal analysis, project administration, validation, writing – original draft, writing – review and editing. **Michelle Bockmann:** conceptualization, methodology, project administration, resources, writing – review and editing. **Alan Brook:** conceptualization, methodology, project administration, supervision, writing – review and editing. **Angela Gurr:** investigation, methodology, resources, validation, writing – review and editing. **Lyle Palmer:** methodology, supervision, writing – review and editing. **Toby Hughes:** conceptualization, investigation, methodology, formal analysis, funding acquisition, project administration, resources, supervision, writing – review and editing.

## Funding

This work was supported by the National Health and Medical Research Council (APP157904), Australian Dental Research Foundation (08/2012) and University of Adelaide Paul Kwok Lee Bequest (PKL 75131608, PKL 75131612 and PKL 75134783).

## Ethics Statement

Ethical approval was granted by the Human Research Ethics Committee of the University of Adelaide (Reference number: H‐2023‐060).

## Consent

The authors have nothing to report.

## Conflicts of Interest

The authors declare no conflicts of interest.

## Supporting information


**Figure S1:** Thin plate spline (TPS) grids illustrating the average deformation of dental arches across different dentition stages for the second principal component (PC2).

**Figure S2:** Thin plate spline (TPS) grids illustrating the average deformation of dental arches across different dentition stages for the third principal component (PC3).

**Table S1:** Comparison of mean arch shape between males and females.
**Table S2:** Comparison of mean arch shape between monozygotic (MZ) and dizygotic (DZ) twins.

## Data Availability

The data that support the findings of this study are available from the corresponding author upon reasonable request.
