# Supplementary material for: Genetic and Environmental Influences on Dental Arch Shape During Development: A Longitudinal Twin Study
Source: Orthod Craniofac Res. 2026 Jul 1;29(6):1134–43. doi: 10.1111/ocr.70162 (PMC13752611; doi:10.1111/ocr.70162)
Supplement: Supplementary file 1 [file OCR-29-1134-s001.docx]

**Supplementary Table S1: Comparison of mean arch shape between males and females**

| **Arch shape comparison** | **Dentition stage** | **Goodall’s F-test** | |
| --- | --- | --- | --- |
|  |  | **F** | **P value** |
| Male maxillary to female maxillary | Primary | 1.97 | 0.062 |
| Male mandibular to female mandibular |  | 1.64 | 0.104 |
| Male maxillary to female maxillary | Mixed | 2.27 | 0.016* |
| Male mandibular to female mandibular |  | 1.81 | 0.043* |
| Male maxillary to female maxillary | Permanent | 3.54 | 0.002* |
| Male mandibular to female mandibular |  | 2.61 | 0.015* |

*P value < 0.05 (statistically significant difference between the groups)

**Supplementary Table S2: Comparison of mean arch shape between monozygotic (MZ) and dizygotic (DZ) twins**

| **Arch shape comparison** | **Dentition stage** | **Goodall’s F-test** | |
| --- | --- | --- | --- |
|  |  | **F** | **P value** |
| MZ maxillary to DZ maxillary | Primary | 0.81 | 0.550 |
| MZ mandibular to DZ mandibular |  | 1.45 | 0.144 |
| MZ maxillary to DZ maxillary | Mixed | 1.19 | 0.225 |
| MZ mandibular to DZ mandibular |  | 1.47 | 0.097 |
| MZ maxillary to DZ maxillary | Permanent | 1.37 | 0.165 |
| MZ mandibular to DZ mandibular |  | 0.99 | 0.369 |

*P value < 0.05 (statistically significant difference between the groups)
